# Direct Preparation of Carbon Nanotube Intramolecular Junctions on Structured Substrates

**DOI:** 10.1038/srep38032

**Published:** 2016-12-01

**Authors:** Jianing An, Zhaoyao Zhan, Gengzhi Sun, Hari Krishna Salila Vijayalal Mohan, Jinyuan Zhou, Young-Jin Kim, Lianxi Zheng

**Affiliations:** 1 School of Mechanical and Aerospace Engineering, Nanyang Technological University, 50 Nanyang Avenue, 639798, Singapore; 2 Chongqing Institute of Green and Intelligent Technology, Chinese Academy of Sciences, Chongqing, 401122, P. R. China; 3 Key Laboratory of Flexible Electronics & Institute of Advanced Materials, Jiangsu National Synergetic Innovation Center for Advanced Materials, Nanjing Tech University, 30 South Puzhu Road, Nanjing, 211816, P. R. China; 4 School of Physical Science and Technology, Lanzhou University, Lanzhou, 730000, P. R. China; 5 Department of Mechanical Engineering, Khalifa University of Science, Technology and Research, Abu Dhabi, 127788, United Arab Emirates

## Abstract

Leveraging the unique properties of single-walled carbon nanotube (SWNT) intramolecular junctions (IMJs) in innovative nanodevices and next-generation nanoelectronics requires controllable, repeatable, and large-scale preparation, together with rapid identification and comprehensive characterization of such structures. Here we demonstrate SWNT IMJs through directly growing ultralong SWNTs on trenched substrates. It is found that the trench configurations introduce axial strain in partially suspended nanotubes, and promote bending deformation in the vicinity of the trench edges. As a result, the lattice and electronic structure of the nanotubes can be locally modified, to form IMJs in the deformation regions. The trench patterns also enable pre-defining the formation locations of SWNT IMJs, facilitating the rapid identification. Elaborate Raman characterization has verified the formation of SWNT IMJs and identified their types. Rectifying behavior has been observed by electrical measurements on the as-prepared semiconducting-semiconducting (S-S) junction.

In modern electronics, junctions are serving as one of the key components, by both providing reliable interconnections and acting as functional building blocks. In traditional electronics, however, these junctions are in the scale of micrometer, which limits the device density and hinders the miniaturization of electronic circuits. In view of this, intramolecular junctions (IMJs) – the junctions existing in single molecules, are the promising candidates for functional elements in molecular electronics., Their development has been regarded as the ultimate path to drive the miniaturization beyond the limits of integrated circuits^1^,^2^.

Among all forms of IMJs, the ones based on single-walled carbon nanotubes (SWNTs) have been most widely and intensely studied^3^,^4^. SWNTs are one-dimensional macromolecular systems that possess superior electronic, physical, chemical, and mechanical properties^5^. In particular, their high carrier mobility^6^, high on-off ratio^7^, and high current carry capacity^8^ render them attractive candidates for achieving high-performance nanoelectronics. A wide range of electronic applications have been proposed, including field-effect transistors (FETs)^9^, ultra-sensitive chemical- & bio-sensors^10^,^11^, interconnects^12^, and transparent conductive membranes^13^. Depending on the diameter and chirality, SWNTs can be classified as either metallic or semiconducting tubes^14^, which show distinct electrical transport behavior. Metallic SWNTs possess high carrier mobility, which is equivalent to that of highly conductive metals, suggesting that they would make ideal interconnects in nanoelectronics. At the same time, the intrinsic characteristics of the semiconducting SWNTs, as controlled by their topology, allow us to build functional devices in nanometer scale^15^. In this regard, there is great interest in fabricating SWNT-based junctions with different structures and properties, by seamlessly joining two or more SWNT segments together.

In spite of great potential of SWNT IMJs, there remain tremendous challenges that hamper their wide applications, and one of the challenges is their controllable synthesis. IMJs could be formed from intrinsic topological defects generated during the conventional SWNT growth, but unfortunately those topological defects are randomly distributed in SWNTs and hard to be identified, not to mention controlling their effects on the properties of SWNTs. Various other synthesis methods have also been proposed, including electron or ion irradiation^16^, e-beam welding^17^, and chemical^18^ & electrostatic^19^ doping. Again, most of those IMJs were obtained by chance, thus it is difficult to fabricate stable SWNT IMJs in a controllable and high-yield manner. In addition, identifying or characterizing the as-obtained IMJs in an accessible way has been regarded as another significant challenge in studying SWNT IMJs. Traditional characterization tools, such as scanning electron spectroscopy (SEM) and atomic force microscopy (AFM), relatively lack high resolution requested for the identification of the topological defects in SWNTs. The atomic structure of SWNT IMJs has been directly imaged by scanning tunneling microscopy (STM)^20^ and high-resolution transmission electron microscopy (HRTEM)^21^, but the low mass elements severely limit the availability of these two instruments in rapid identification of SWNT IMJs over a large length scale. Most recently, resonance Raman spectroscopy, a technique probing the vibrational properties of the nanotubes, has been proven to be a powerful tool in SWNT research^22^. It is much more convenient compared to STM and HRTEM, because it is non-contact and non-destructive, can be operated at room temperature under ambient environment, and is capable of providing valuable insights into the diameter and chirality of a single nanotube. However, when Raman spectroscopy alone is used to study isolated SWNTs, especially for characterizing the SWNT IMJs, the process is time-consuming due to Raman’s narrow resonance window.

In this paper, we present an advanced strategy to controllably prepare SWNT IMJs on a large scale. Our approach involves growing ultralong SWNTs *via* a chemical vapor deposition (CVD) process on trenched substrates to intentionally induce strains and bending deformation in individual SWNTs. Since the CVD growth of ultralong and well-oriented SWNTs follows the empirical “floating growth” mode^23^, the SWNTs are capable of flying over the patterned substrate and eventually lying suspended across a series of trenches. These trenches will introduce designed strains in the partially suspended nanotubes and induce mechanical deformations, which will locally distort the nanotubes’ lattice and modify their electronic structure, resulting in the formation of SWNT IMJs. The trenches also define the as-prepared IMJs’ locations – in the vicinity of the trench edges. By taking the trenches as markers, we utilized Raman spectroscopy to rapidly and precisely identify the SWNT IMJs and comprehensively investigate their properties.

## Results

### Preparation of SWNT IMJs

**Figure 1:**
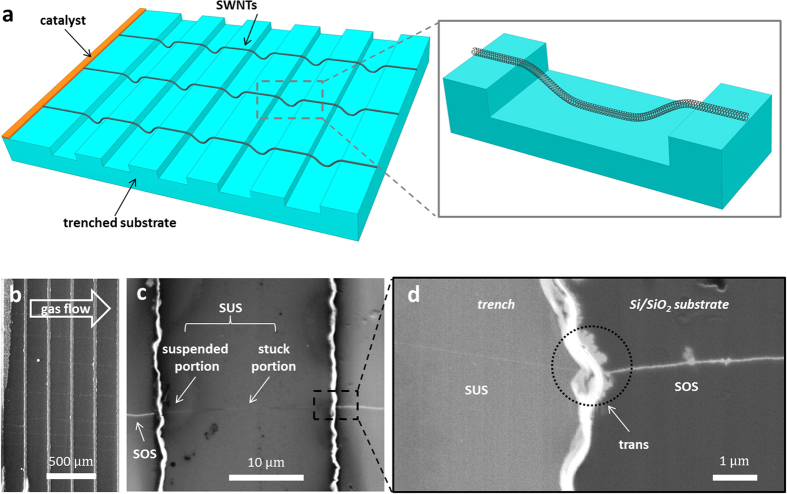
Preparation of SWNT IMJs through controlled CVD process. (**a**) Schematic diagram showing the preparation of SWNT IMJs. The magnified view clearly shows an individual SWNT lying across a trenched structure, the middle segment of the nanotube is in contact with the trench bottom. (**b**) SEM image showing oriented SWNTs grown on a trenched Si/SiO_2_ substrate. (**c**) An ultralong SWNT lies partially suspended across a 20 μm wide and 2 μm deep trench. Segments of the nanotube are distinguished and indicated in the image. (**d**) Zoom-in SEM observation of the trench edge; the trans segment of the tube is highlighted with black dot circle.

Figure 1a schematically illustrates the preparation process of SWNT IMJs. The Si/SiO_2_ substrate for SWNT IMJs synthesis was patterned with 20 μm wide and 2 μm deep trench stripes; the spacing between two adjacent trenches was 200 μm. After applying the catalyst precursor at one end of the substrate, CVD was carried out to grow ultralong SWNT arrays on the trenched substrate. SEM image in Fig. 1b shows the SWNT arrays stemming from the catalyst region and elongating downstream while crossing the trenches. All SWNTs are lying in parallel; the separation between any two adjacent SWNTs is wider than 100 μm, which is wide enough for carrying out Raman spectroscopy on spatially isolated nanotubes. The high-magnification SEM image (Fig. 1c) shows a representative individual SWNT, which extends over 1 cm in length, spanning across a trenched structure. Due to the small aspect ratio (ratio between the height and the width) of the trench, the SWNT lies partially suspended on top of the trench, while its middle portion contacts with the trench bottom. Thus, the individual nanotube can be clearly identified as three different segments: the sitting-on-substrate segment (SOS), the transition segment (trans, in the vicinity of the trench edge), and the suspended segment (SUS) that consists of the suspended portion and the stuck portion, as indicated in the SEM images. Figure 1d shows the trench edge region with a higher magnification, from where the trans segment can be clearly identified. Due to the different charging environment^24^, the SOS segment appears brighter and wider than the SUS segment at the acceleration voltage of 1 kV.

### Characterization of individual SWNTs

To validate that IMJs have been generated in the SWNTs, it is important to make sure that we are handling with isolated SWNTs instead of thin bundles, and the studied SNWTs are relatively defect-free. Because only after eliminating other possibilities such as tube-tube interaction that may affect the tube structure and properties, we can attribute the changes in tube structure and properties to the formation of IMJs. Thus, the structure and properties of the as-grown SWNTs were carefully characterized using Raman spectroscopy in the first place.

**Figure 2:**
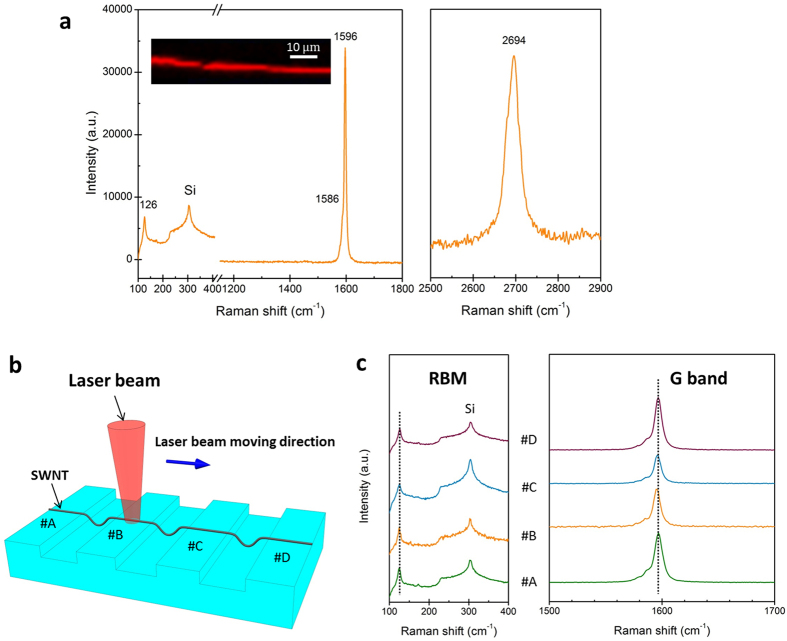
Raman characterization of SOS segments of an individual SWNT. (**a**) Typical G band map (inset) of the SOS segment of one isolated as-grown SWNT, with 514 nm excitation wavelength. A representative Raman spectrum acquired from a single spot of the SOS segment exhibits strong Raman features of RBM, D band, G band, and G’ band. (**b**) Schematic diagram of Raman characterization of different SOS segments of the specific SWNT. (**c**) Evolution of Raman features along the nanotube length. The series of Raman spectra were acquired as the 514 nm laser beam moving along the nanotube axis. The labels (#A-#D) indicate different locations on the Si/SiO_2_ substrate, referring to (**b**).

At single nanotube level, resonance Raman measurements were performed on the SOS segments of a representative ultralong SWNT (longer than 1 cm), with 514 nm laser excitation. From the Raman map that was obtained from the integrated intensity around the G band (see the inset of Fig. 2a), the SWNT can be spatially resolved. To study its characteristics, Raman spectrum was collected from a single spot (more than 1 mm away from the catalyst region) of the nanotube, as shown in Fig. 2a. The Raman spectrum exhibits a single radial breathing mode (RBM) centered at 126 cm^−1^, with a linewidth (full width at half maximum, FWHM) of 8 cm^−1^, we thus obtained *d*_*t*_ of 1.97 nm by using the correlation *d*_*t*_ = 248/*ω*_RBM_ for nanotubes grown on Si/SiO_2_ substrates^25^. By comparing with the Kataura plot, this nanotube is tentatively assigned to the (22, 5) semiconducting tube^26^, whose optical transition energy 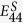 is in resonance with the 2.41 eV laser excitation energy. The intense G band of the nanotube displayed in Fig. 2a can be fitted by two Lorentzians, with one peak at 1596 cm^−1^ (FWHM = 7 cm^−1^) for the G^+^ component and the other peak at 1586 cm^−1^ (FWHM = 9 cm^−1^) for the G^−^ component. This G band feature further corroborates the semiconducting nature of the nanotube^27^, which is consistent with the RBM assigning. Besides, the narrow linewidths of the two G band components indicate that the Raman spectrum is taken from an isolated SWNT rather than a thin bundle^28^. D band is hardly observed in the Raman spectrum, which implicates the high quality of the examined nanotube^29^. Moreover, a single sharp and symmetric Lorentzian peak is observed at 2694 cm^−1^ (FWHM = 34 cm^−1^), which can be assigned to the G′ band arising from a double resonance process^30^. The symmetric line shape, narrow linewidth, and high intensity of the G′ band signify that the examined nanotube is an isolated SWNT, rather than a double-walled nanotube (DWNT) or a thin bundle of SWNTs^30^.

In order to provide additional evidence that the examined ultralong nanotube is an isolated SWNT, we further mapped the nanotube using SEM and AFM over a large area; only one nanotube was observed in both SEM and AFM measurements. Tapping mode AFM imaging was performed on an SOS segment for a total length of 200 μm; the AFM image (Supplementary Fig. S1a) shows a 30 μm portion of the probed SOS segment. A detailed image of the nanotube was obtained by a zoom-in scan (see the magnified AFM image of Supplementary Fig. S1b). The corresponding height profile (Supplementary Fig. S2c) indicates the diameter of the nanotube is around 1.9 nm, consistent with our Raman calculation.

### Chiral and dimensional uniformity

The probed isolated SWNT (longer than 1 cm) lies partially on the Si/SiO_2_ substrate spanning across plenty of 20 μm wide and 2 μm deep trenches along its length, as sketched in Fig. 2b, which allows us to evaluate its chiral and structural uniformity. A series of Raman spectra were acquired at different on-substrate locations (denoted as *i.e.* A, B, C, and D, in Fig. 2b), as the laser beam was scanned along the nanotube axis. The corresponding evolutions of the Raman features are presented in Fig. 2c. Both the RBMs and the G bands maintain their wavenumbers and line shapes at all spots, indicating a high degree of chiral and structural uniformity along this SWNT. The AFM height profiles acquired along the tube length also show a high consistency in the nanotube diameter (1.85~1.95 nm), which provides additional indication of a uniform individual SWNT^31^. The good chiral and dimensional uniformity of the nanotube along its length offers the possibility of controllable creation of SWNT IMJs in desired locations.

### Identification and characterization of SWNT IMJs

**Figure 3:**
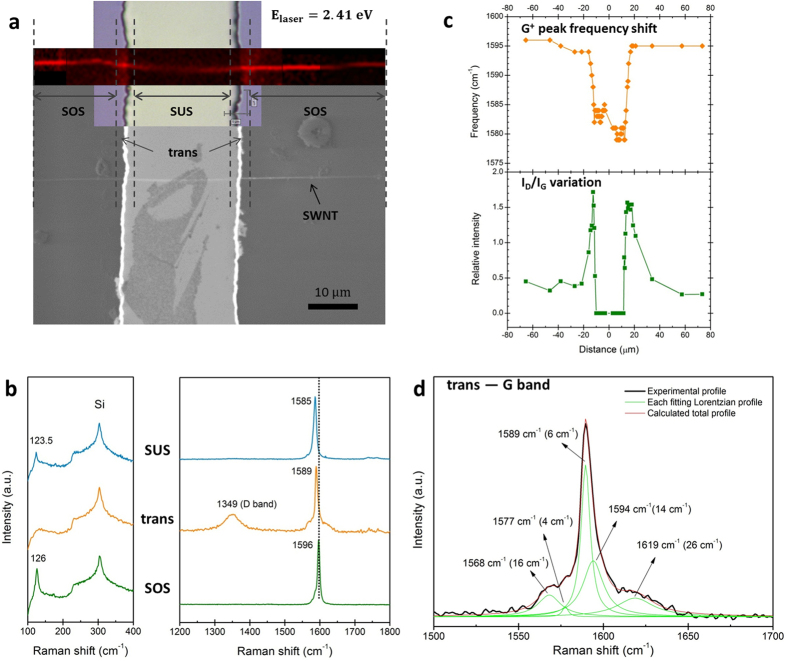
Identification and characterization of SWNT IMJs. (**a**) Raman map of the (22, 5) nanotube lying partially suspended on one 20 μm wide and 2 μm deep trench. The optical image shows the geometric configuration of the trench. SEM image provides additional spatial information about the nanotube and the trench. The three regions can be clearly differentiated from the Raman map and SEM image, as respectively indicated as “SOS”, “trans”, and “SUS”. (**b**) Comparison of Raman features obtained from SOS, trans, and SUS segments respectively, showing evolutions through the three regions (*E*_laser_ = 2.41 eV). (**c**) 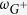 (top panel) and *I*_*D*_/I_*G*_ ratio (bottom panel) versus positions along the axis of the nanotube across a trench. The middle point of the trench (along the trench width) is set as zero, the positions at “−10 μm” and “10 μm” imply the two edges of the trench. (**d**) Specific analysis of the G band taken from the trans segment, using Lorentzian function fitting. The experimental profile (black line), each fitting Lorentzian profile (green line), and the calculated total profile (red line) are displayed respectively.

We continuously mapped the (22, 5) nanotube across a 20 μm wide and 2 μm deep trench, using the laser excitation of *E*_laser_ = 2.41 eV. Figure 3a shows a Raman image (generated by G band intensity) of the partially suspended SWNT superimposed on an optical image where the trenched structure is visible. The corresponding SEM image provides additional spatial information about the nanotube and the trench. The aforementioned “SOS”, “trans”, and “SUS” segments are clearly discernible from the substrate to the trench along the tube length.

Guided by this Raman image, Raman spectra were acquired from each region and displayed in sequence for comparison (Fig. 3b). The RBM shows a frequency downshift by 2.5 cm^−1^ from SOS segment to SUS segment, while no obvious RBM signal is detected from the trans segment. This downshift in RBM frequency is in agreement with the previous reports^32^,^33^, which is attributed to the radial deformation of the nanotube induced by tube-substrate interactions by distorting the nanotube cross section from a circle to an ellipse. In addition, the linewidth of the RBM is reduced significantly in the SUS segment (5 cm^−1^) compared with the SOS segment (8 cm^−1^), which is expected to be the result of a relatively undisturbed environment for the SUS segment^34^. Since the RBM is highly sensitive to the resonance condition, the loss of RBM in the trans segment suggests a structural change, which turns the nanotube into “off resonance” state with the laser excitation. Based on the drastic evolution of the RBMs in the three regions, we speculate that an IMJ may have been formed in the trans segment.

The higher-frequency Raman features also undergo a drastic change through the three segments. The G band of the SOS segment shows a semiconducting line shape, with G^+^ peak at 1596 cm^−1^ and G^−^ peak at 1586 cm^−1^, respectively (Fig. 3b, right). As the laser beam moves towards the trench, although the nanotube sustains its semiconducting characteristic, the frequency of G^+^ band downshifts from 1596 cm^−1^ for the SOS segment to 1589 cm^−1^ for the trans segment and further to 1585 cm^−1^ for the SUS segment. Similar frequency downshift is observed from G^−^ band as well–from 1586 cm^−1^ (SOS) to 1577 cm^−1^ (trans) and further to 1567 cm^−1^ (SUS). Potential factors, such as doping, thermal effect, and strain, may result in continuous frequency downshift in G band^35^,^36^,^37^. First of all, the doping effects, including intentional doping, contaminant, or electron transfer from the substrate, do not have significant influence on such large amount of downshifts as demonstrated in our previous work^38^. Secondly, in order to examine the possible thermal effect induced by laser illumination, we performed a set of Raman imaging (*E*_laser_ = 2.41 eV) of an individual SWNT lying across a trenched structure (20 μm wide and 2 μm deep) at different laser power levels under identical other conditions. The results suggest that the laser power effect is negligible in our work (see Supplementary Discussion and Supplementary Fig. S2). The third possibility of the frequency downshift is due to the “self-built” tensile strain, existing in the SWNTs decorated with graphite dots^39^. However, there was no graphite dot decorated on our SWNT, as observed in the magnified AFM and SEM images; this verifies that the so-called “self-built” tensile strain does not exist in our SWNT sample. Therefore, strains induced by the trenched structure should be accounted for the frequency downshift of G band. This is very reasonable, because as reported previously, the G^+^ peak frequency could be pushed down by 40 cm^−1^ to lower frequency under a strain of 1.65%^40^. The effect of strain on the G^+^ peak frequency was monitored in different locations along the tube axis. As shown in the upper panel of Fig. 3c, 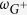 stays almost invariant for SOS segment, falls precipitously within the trans region (2~3 μm away from the trench edge at both sides), and then plateaus with very small fluctuations (<2 cm^−1^) for the SUS segment. This suggests that the SWNT was drastically stretched by the strain, resulting in elongation in the C-C bonds along tube axis and reduction of the bond energy.

We further investigated the detailed G band feature obtained from the trans segment. The G band of the trans segment can be fitted by five Lorentizian peaks (Fig. 3d), unlike the G band profiles of the SOS and SUS segments, which only consist of two components. Previous studies reported that due to the nanotube curvature effects, up to six G modes are Raman allowed in SWNTs^27^. In particular, polarized Raman studies of SWNTs revealed that, for semiconducting tubes, the G band profile can be de-convolved into four intrinsic components with the following symmetries: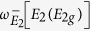, 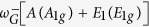, 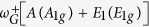, and 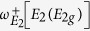
41. Thus, we can assign the two peaks located at 1589 cm^−1^ (6 cm^−1^) and 1577 cm^−1^ (4 cm^−1^) to the G^+^ and G^−^ modes, respectively. The two wide shoulders on the low-frequency side (1568 cm^−1^ (16 cm^−1^)) and high-frequency side (1594 cm^−1^ (14 cm^−1^)) are assigned to 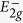 and 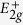 phonon modes, respectively. The appearance of new G components suggests that, in addition to uniaxial strain, bending deformation may also have been induced in the SWNT at the edge of the trench (trans segment region). Because the uniaxial strain does not change the numbers of Raman modes^42^, while the predominant effect of the bending deformation on Raman spectra of SWNTs is to increase the number of active Raman modes^43^. Intriguingly, a new component at ~1619 cm^−1^ (26 cm^−1^) emerges as a shoulder of the G profile in this region, which is normally termed as D′ band in graphitic materials^44^. It is well established that the D′ band is a defect-induced double resonance feature in CNTs^45^. As a consequence, its appearance implies the presence of disorders and defects in the trans segment.

Another striking observation is that a strong D band situated at 1349 cm^−1^ is activated in the Raman spectrum taken from the trans segment (Fig. 3b). D band comes from second-order double resonance processes and becomes prominent when the nanotube’s lattice symmetry is broken by disorders and defects^46^. By taking the intensity ratio of D band and G band (*I*_*D*_/*I*_*G*_) as the fingerprint for disorders in SWNTs, we plotted the *I*_*D*_/*I*_*G*_ ratio along the tube axis, as shown in Fig. 3c lower panel. The *I*_*D*_/*I*_*G*_ ratio ascends from SOS segment to trans segment, culminates in the trans region, and then descends to zero in the SUS segment. The disappearance of D band of the SUS segment implies this nanotube is intrinsically defect-free. The pronounced D band of the trans segment signifies that disorders and defects have been generated in the trans segment of the SWNT, which is consistent with the analyses of the G band feature of trans segment. Thus we can conclude that a junction has been formed between the SOS and SUS portions, as a result of the mechanical deformation in this region^4^. In addition, by reviewing the transition in the line shape of the G band along the nanotube (from SOS to SUS), we identified this IMJ as a semiconducting-semiconducting (S-S) junction. This IMJ also exhibits an asymmetric nonlinear feature in current-voltage (*I*−*V*) curve (Fig. S4), similar to that of a rectifying diode.

## Discussion

It is well accepted that ultralong SWNTs float above the substrate during the growth and then settle down at a later time^47^. In the presence of trenched structures on the substrates, as SWNTs adhere to the contours of the substrate, they are zipped at the trench edges, due to the height difference between the top flat surface and the trench bottom; this “zipping motion” induces tensile strain in the SWNTs^38^. Such an induced strain is only about 0.5% calculated from Raman shift^38^,^40^. Besides, because of the trench geometry, the middle portion of the SUS segment is bound to the surface due to the strong adhesion energy^48^. A previous study predicted the tremendous axial strain induced by the van der Waals interaction between the stuck tube portion and the trench bottom^49^. Based on the established model, we were able to estimate that a strain of 0.9% has been induced in our probed nanotube portion (see Supplementary Information for detailed calculation). In either case, the strain is far below the threshold of SWNT’s plastic deformation^50^, thus is not the only reason for the generation of disorders in the tube lattice.

**Figure 4:**
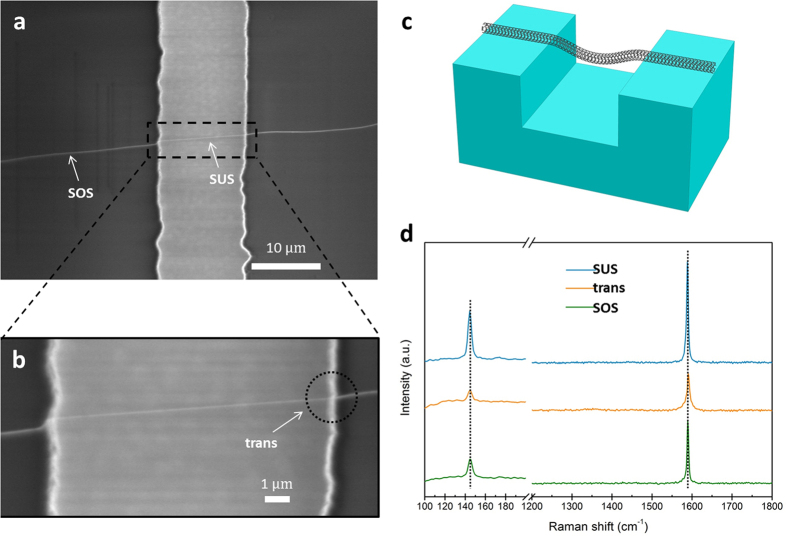
Individual SWNT lying fully suspended across a narrow and deep trench. (**a**) SEM image of an individual SWNT growing across a 10 μm wide and 10 μm deep trench. (**b**) Magnified view showing the nanotube is fully across the trench, without touching the trench bottom. (**c**) 3D schematic configuration of the fully suspended SWNT. (**d**) Evolution of Raman spectra obtained from the three segments. Active Raman modes (RBM and G band) respectively sustain the frequency almost un-shifted and feature line shape un-changed.

The formation mechanism of IMJs was then explored by using different trenched structures. We intentionally grew an ultralong SWNT across a 10 μm wide and 10 μm deep trenched structure (narrower and deeper), as shown in Fig. 4a. A zoom-in SEM image in Fig. 4b reveals the portion inside the trench, which looks perfectly straight and taut, suggesting that the nanotube is fully suspended across the trench without touching the lower substrate (Fig. 4c). With this “narrow and deep” configuration, we ensured that the nanotube is solely subject to strains induced by the “zipping effect” at the trench edges. Raman spectra were respectively collected from the three segments with 514 nm laser excitation and are displayed in Fig. 4d. First of all, the RBM at 144.4 cm^−1^ neither shifts its frequency nor changes its profile with the move of laser spot position along the tube axis. Secondly, from SOS to trans further to SUS, the G band sustains its line shape, while 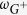 only downshifts by 2 cm^−1^ from SOS (at 1590 cm^−1^) to SUS (at 1588 cm^−1^). Finally, the D band is invisible in all these three spectra, even in the spectrum taken from the trans segment. From a detailed statistic study by varying the trench width from 10 μm to 100 μm and the trench depth from 1 μm to 10 μm, we always found the successful formation of IMJs for partially suspended SWNTs, but there was no obvious change in line shape for fully suspended SWNTs, even occasionally large Raman shift (~10 cm^−1^) were observed^38^.

Therefore we can now conclude that the axial strain together with the bending deformation enables the formation of IMJs. The axial strain propagates along the tube axis, drags the suspended portion downwards, and thereby promotes the bending deformation of the SWNT at the edge of the trench. As a result, the outer side of the nanotube segment is stretched while its inner side is compressed, which is in analogous to the scenario of using an AFM tip to push a suspended SWNT^51^. The resultant bending angle was around 16° (equal to the contact angle in magnitude). Under such large bending deformation, the local lattice symmetry of the nanotube is severely distorted because the C-C bonds are elongated for the outer side and shortened for the inner side. Then a transition from sp^2^ to sp^3^ in the bonding configuration occurs in the local bending region^51^. The localized disorders further affect the density of states (DOS) at the Fermi level, thus tuning the band gap of the nanotube and forming a junction in the deformation region^4^,^52^. The proposed formation mechanism has been further validated by studying other trenched structures, where the middle portion of the spanning SWNT is in contact with the trench bottom. Given that 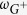 shift and *I*_*D*_/*I*_*G*_ ratio variation provide great information in identifying the as-fabricated IMJs, we have explored the trend of these two factors along the tube axis. As seen from the four typical results exemplified in Supplementary Fig. S5, 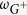 always starts downshifting and *I*_*D*_/*I*_*G*_ ratio always reaches its peak value in the trans region, indicating the universality of SWNT IMJs formation at the trench edges. In addition, the amount of strain and extent of bending deformation induced to the SWNT can be simply adjusted by varying the geometrical parameters of the trenched structures, which points out the possibility of forming IMJs with diverse mechanical and electrical properties in one-batch production^53^,^54^.

In summary, we have demonstrated a simple, straightforward, and reliable method for controllable preparation of SWNT IMJs. This has been asserted by growing ultralong SWNTs on trenched Si/SiO_2_ substrates. With a combination of SEM, AFM, Raman spectroscopy, and electrical measurement, the formation of SWNT IMJs has been verified. Elaborate characterizations of the IMJs attributed the formation mechanism to the axial strain and bending deformation. The trenched structures employed here possess tremendous advantages. Firstly, they provide a feasible, controllable, and robust way of synthesizing SWNT IMJs. Secondly, the geometry of the trench defines specific formation location, facilitating the identification and characterization of the IMJs. Finally, the structures and properties of the IMJs can be modulated by adjusting the geometrical parameters of the trenches, promising an approach to fabricate different types of IMJs at the same time. Our research into the IMJs is directed toward fostering the fabrication of innovative nanodevices and improving the integration of nanoelectronics.

## Methods

### Fabrication of trenched structures on flat Si/SiO_2_ substrate

Stripe patterns were firstly developed on the surface of Si/SiO_2_ substrates using a standard photolithographic technique, where the 1 μm of SiO_2_ layer was thermally grown on the 4 inch Si wafer. In the following step, trenched structures were created by using deep reactive ion etching (RIE) process. After removing the photoresist, the trenched substrates are ready for SWNT growth. The width of the trenched structures was recorded from the mask design, while the depth could be increased from 1 to 10 μm by elongating the etching time.

### Synthesis of SWNT IMJs

By applying catalyst precursors on the top surface of the as-fabricated trenched substrates, CVD process was carried out to grow ultralong SWNTs lying across trenches, for the purpose of controllably preparing IMJs (Fig. 1a). To introduce strains and generate defects in isolated nanotubes instead of thin bundles, sparse SWNT arrays with wide separation are better candidates in this study. As discussed in our previous work^55^, the density of the SWNT arrays can be greatly affected by the growth conditions. In this regard, we set the growth recipe as: 0.01 M FeCl_3_ ethanol solution serving as the catalyst precursor was reduced to Fe nanoparticles under 120 sccm of Ar and 30 sccm of H_2_ gas mixture at 950 °C and ambient pressure. After 20 min of reduction, the growth of SWNTs was triggered by introducing the carbon source (20 sccm of ethanol vapor, bubbled by Ar stream at 20 °C) into the gas flow, while switching Ar and H_2_ to 4 sccm and 6 sccm simultaneously. The growth process continued for 40 min at 950 °C, and eventually the furnace was cooled to room temperature under Ar stream.

### Identification and characterization of SWNT IMJs

SEM (Jeol, JSM-7600F) was utilized to reveal the morphology of SWNTs over a large area. SEM images were taken at 1 kV acceleration voltage. AFM (Asylum Research, Cypher AFM) in tapping mode was as well employed to display SWNT morphology in 2D images. The AFM height profile can provide information of the SWNT diameters. Raman images and spectra of SWNTs were collected with a confocal Raman system (Renishaw, inVia Raman microscope) at room temperature, using three laser lines as the excitation source: 633 nm (HeNe, Renishaw, RL633), 514 nm and 488 nm (Ar^+^, Stellar-Ren, Modu laser, LLC). The spectrometer was equipped with a 100× microscope objective (Leica, NA = 0.85) and an X-Y translation stage. Laser beam was focused on the sample through the microscope objective, providing a 1 μm excitation spot size. The laser power on sample (measured by a power meter) was kept below 0.5 mW to avoid the heating effect. Raman imaging and Raman spectra were taken in the backscattering configuration.

## Additional Information

**How to cite this article**: An, J. *et al*. Direct Preparation of Carbon Nanotube Intramolecular Junctions on Structured Substrates. *Sci. Rep.* **6**, 38032; doi: 10.1038/srep38032 (2016).

**Publisher's note:** Springer Nature remains neutral with regard to jurisdictional claims in published maps and institutional affiliations.

## Supplementary Material

Supplementary Information
