# Supplementary material for: Direct Preparation of Carbon Nanotube Intramolecular Junctions on Structured Substrates
Source: Sci Rep. 2016 Dec 1;6:38032. doi: 10.1038/srep38032 (PMC5131317; doi:10.1038/srep38032)
Supplement: Supplementary Information [file srep38032-s1.doc]

Supplementary Information

Direct Preparation of Carbon Nanotube Intramolecular Junctions on Structured Substrates

Jianing An,^1^ Zhaoyao Zhan,^2^ Gengzhi Sun,^3^ Hari Krishna Salila Vijayalal Mohan,^1^ Jinyuan Zhou,^4^ Young-Jin Kim,*^1^ and Lianxi Zheng*^5^

*^1^ School of Mechanical and Aerospace Engineering, Nanyang Technological University, 50 Nanyang Avenue, 639798, Singapore*

*^2^ Chongqing Institute of Green and Intelligent Technology, Chinese Academy of Sciences, Chongqing, 401122, P. R. China*

*^3^ Key Laboratory of Flexible Electronics & Institute of Advanced Materials, Jiangsu National Synergetic Innovation Center for Advanced Materials, Nanjing Tech University, 30 South Puzhu Road, Nanjing, 211816, P.R. China*

*^4^ School of Physical Science and Technology, Lanzhou University, Lanzhou, 730000, P. R. China*

*^5^ Department of Mechanical Engineering, Khalifa University of Science, Technology and Research, Abu Dhabi, 127788, United Arab Emirates*

**Corresponding author.*

*Email address:* [*yj.kim@ntu.edu.sg*](mailto:yj.kim@ntu.edu.sg) *(Y-J. Kim);* [*lianxi.zheng@kustar.ac.ae*](mailto:lianxi.zheng@kustar.ac.ae) *(L. Zheng)*

**1. Supplementary Figures**


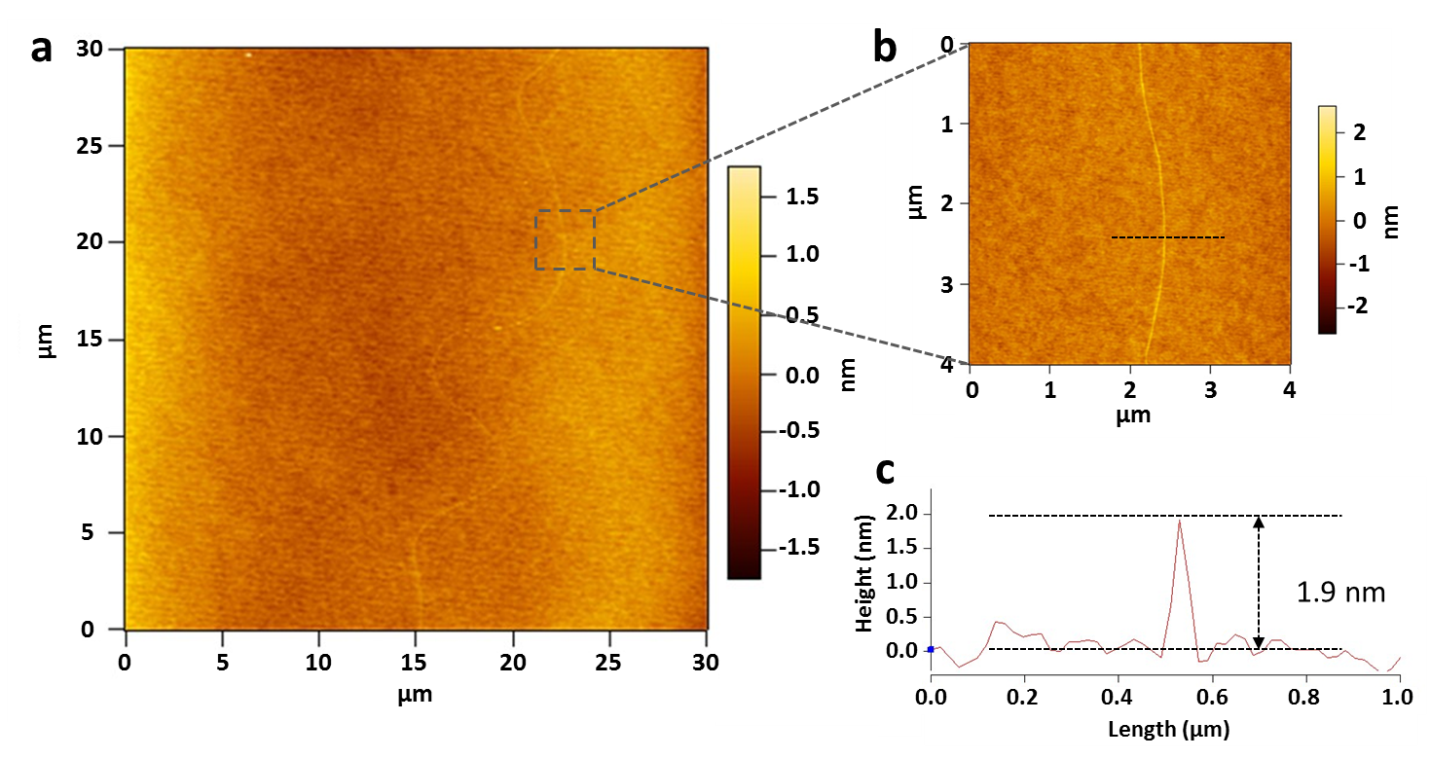


**Supplementary Figure S1 | AFM characterization of an individual ultralong SWNT.** (**a**) Tapping mode AFM map of an SOS segment in a 30 × 30 µm^2^ area. (**b**) Magnified AFM image of the area enclosed by the grey dashed square in (**a**), showing the isolated nanotube. (**c**) Typical height profile of the nanotube indicated in (**b**), indicating the diameter is around 1.9 nm.


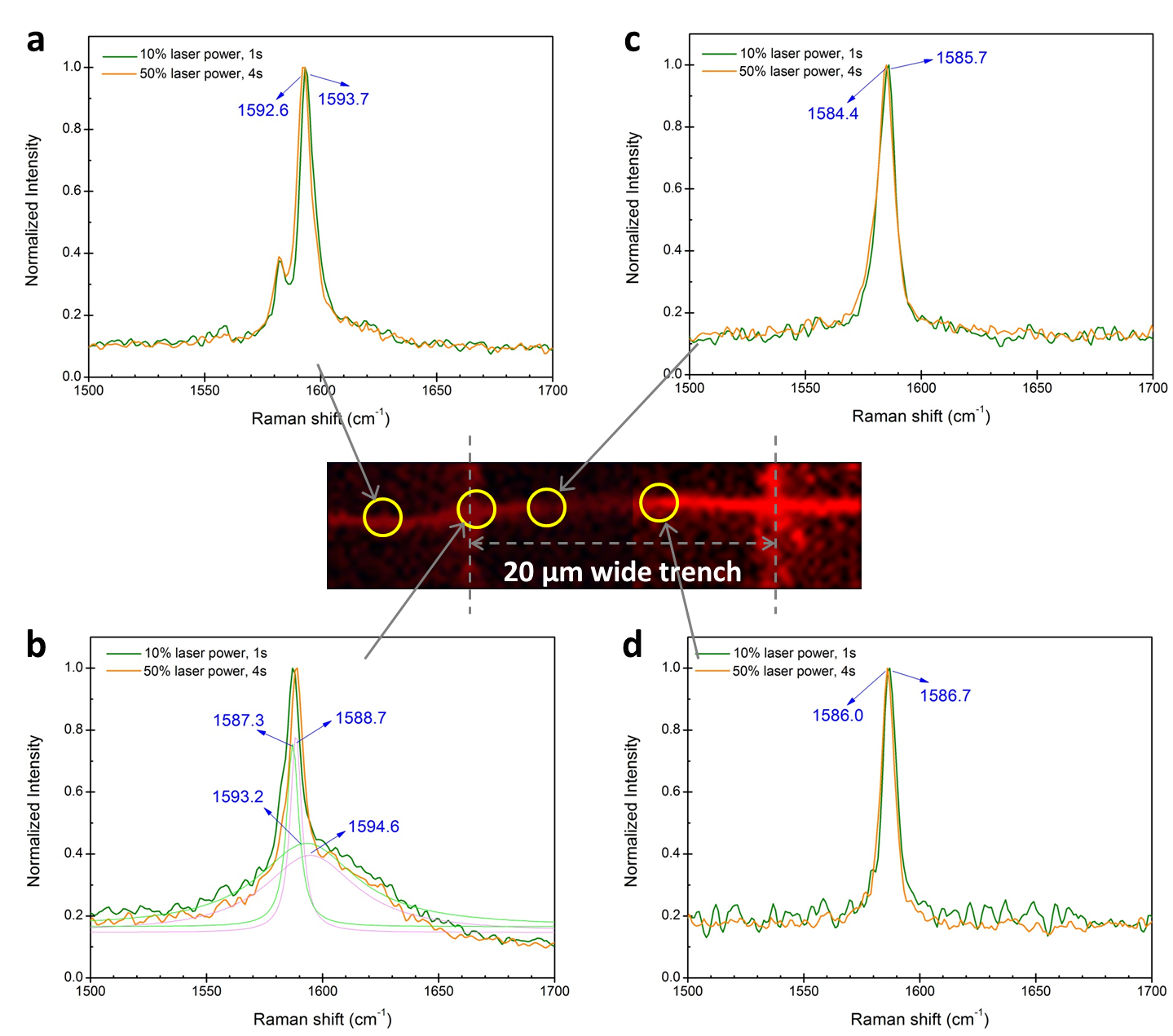


**Supplementary Figure S2 |** **Examination of laser-induced thermal effect on Raman characterization of nanotube.** Raman map (constructed using G peak intensity) showing an individual SWNT lying across a trenched structure. Four spots along the nanotube were picked out for comparing the spectra taken with varied laser energies under same conditions. The corresponding normalized spectra are displayed in (**a**)-(**d**), respectively. The spectra in (**b**) are respectively fitted by two Lorentzian peaks. Aside from the change in the signal intensity, there is no other variation of feature shape and little peak frequency detected in every compared group of spectra.


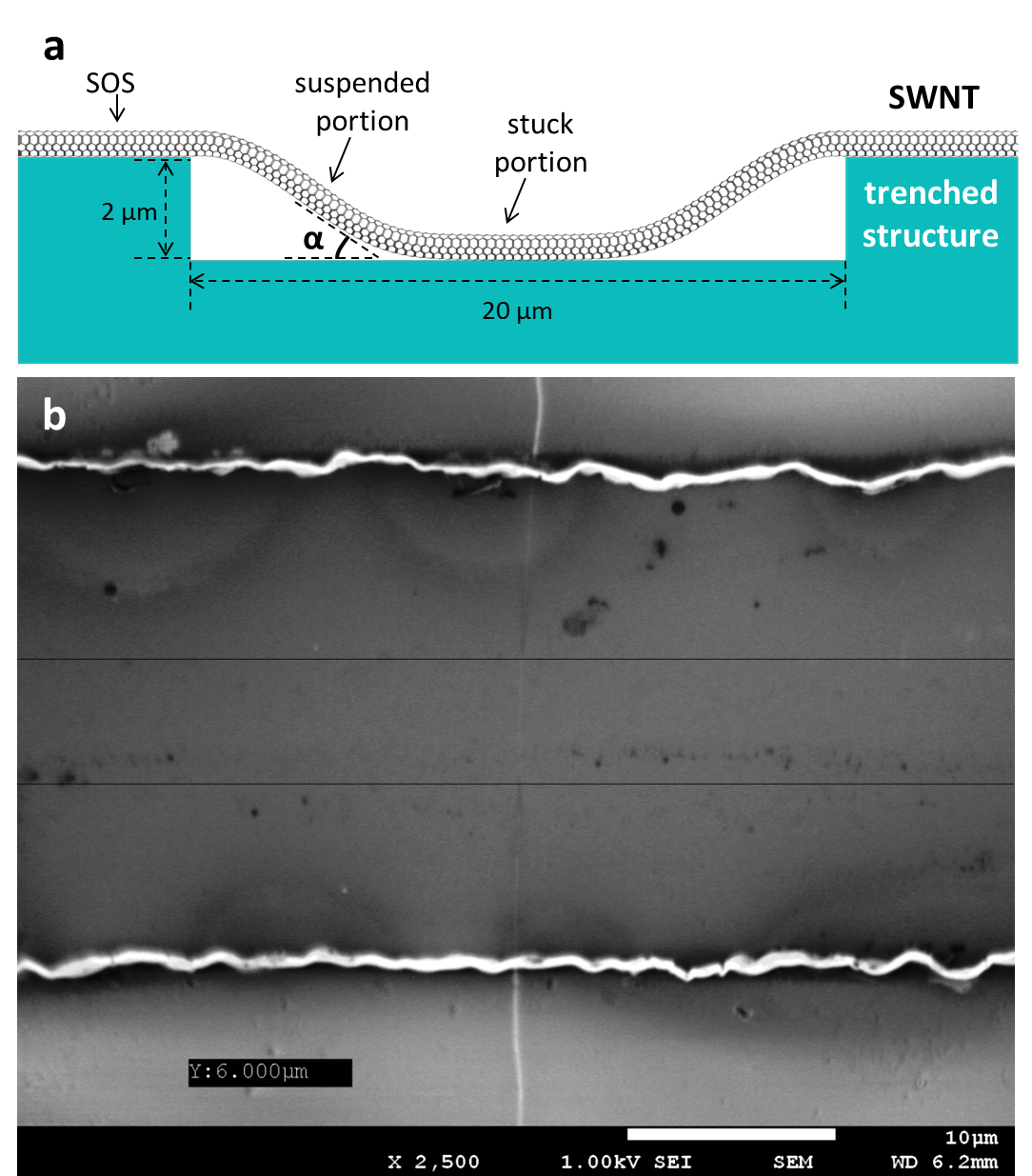


**Supplementary Figure S3 | Calculation of strain induced by van der Waals interaction.** (**a**) A succinct sketch showing the configuration of an individual SWNT lying partially suspended across a trenched structure, with a contact angle $\alpha$. The contact angle can be calculated based on the length of the stuck portion and the trench dimension, as exampled in (**b**).

**
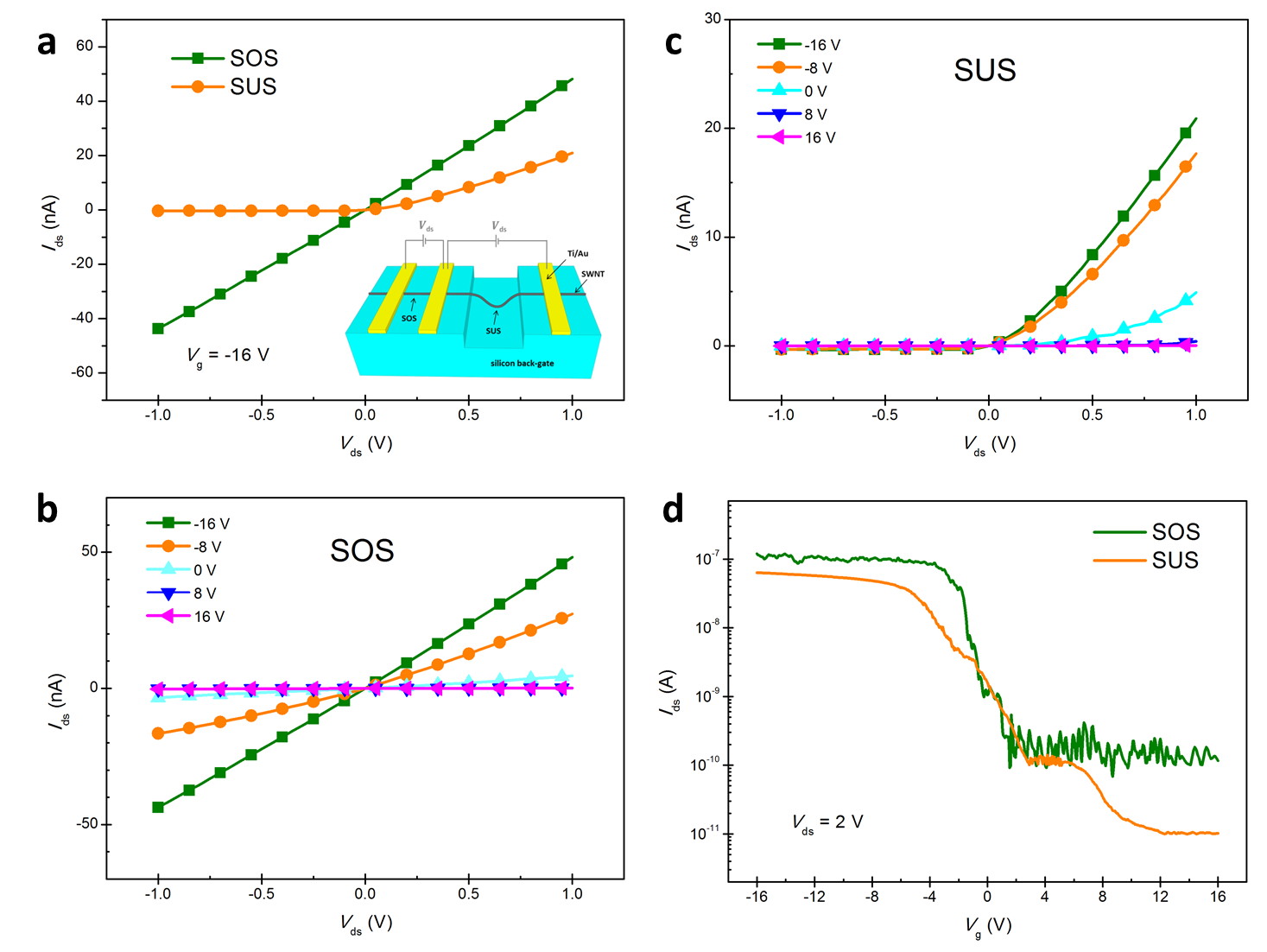
**

**Supplementary Figure S4 | Electrical properties of the parent SWNT (SOS segment) and the SWNT IMJ (SUS segment).** (**a**) Comparison of $I_{\text{ds}}-V_{\text{ds}}$ characteristics of adjacent SOS and SUS segments. The inset shows schematic diagram of the FET devices. (**b**) and (**c**) Variation of $I_{\text{ds}}-V_{\text{ds}}$ curves of the SOS (**b**) and SUS (**c**) segments at different gate voltage levels. (**d**) Plot of $I_{\text{ds}}$ versus $V_{\text{g}}$ of the two segments, under a bias voltage $V_{\text{ds}}$ = 2 V.


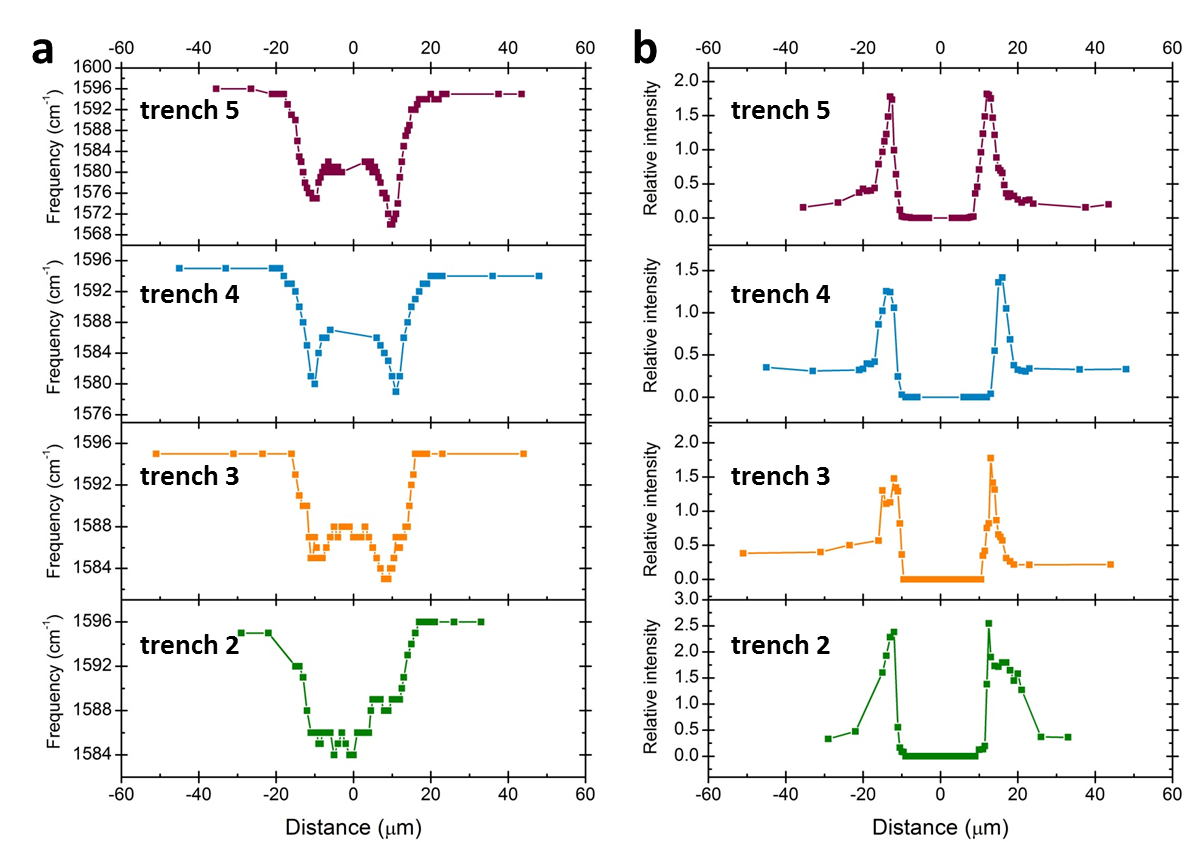


**Supplementary Figure S5 | Study of $\omega_{G^{+}}$ shift and $I_{D}/I_{G}$ ratio variation of nanotubes lying on other trenched structures.** (**a**) $\omega_{G^{+}}$ shift and (**b**) $I_{D}/I_{G}$ ratio variation observed from other SWNT segments crossing wide and shallow trenches.

**2. Supplementary Discussion**

**Laser-induced thermal effect on Raman characterization of SWNTs lying across trenched structures.**

The intensity of the laser used in the Raman characterization experiments should be taken into consideration as a potential factor that results in the observed variation in the Raman features. To probe the possibility, we have Raman mapped an individual SWNT that lies across a wide and shallow trenched structure with varied laser powers under the same condition. The general setting of the Raman imaging is listed here: Grating scan type: static; Center: 1580 cm^-1^; Low: 1373.83 cm^-1^; High: 1781.21 cm^-1^. The laser (514 nm wavelength) power levels with 100% power, 50% power, and 10% power on the sample are respectively 0.322 mW, 0.189 mW, and 0.035 mW, as measured by a power meter (Thorlabs, S121C). All experiments were conducted at room temperature.

The Raman map shown in Fig. S2 gives the information of the studied SWNT lying across the trench. In the first Raman imaging process, we used 10% laser power, with integration time of 1 sec. This setting could provide a sufficient low-intensity laser beam onto the nanotube. In two sets of comparison experiments, we elongated the integration time gradually to 2 sec, 3 sec, and 4 sec, with 10% and 50% laser power respectively to map the same nanotube. In all cases we could observe the same trend of G band frequency shift along the nanotube, in other words, the Raman spectrum is almost invariant by changing the laser power. Here we pick out four typical spots on the nanotube to exhibit the invariant Raman spectra. As shown in Fig. S2a-d, we compared the Raman spectra acquired under two conditions—10% laser power + integration time of 1 sec and 50% laser power + integration time of 4 sec. Due to the difference of the laser power and integration time, the intensity of the signals were altered, therefore in order to directly compare the line shape and frequency of the spectra, the intensities of all spectra were normalized with respect to the maximum height of their G peak. It can be seen that for every investigated spot, there is no change regarding the G feature line shape between the two Raman spectra taken with different laser energies. The possible laser heating effect only induced slight downshift of G peaks (within 1.5 cm^-1^), which is consistent with the reported phenomenon[^1^](#_ENREF_1). This excludes the possibility of thermal breakage of the investigated nanotube.

Thus we can conclude that the laser power is not an influential factor (by irradiating heat) that could induce the change of Raman features, and the observed variations in the Raman spectra (shown and discussed in the main text) should result from other possibilities.

**Calculation of strain induced by the van der Waals interaction**

We adopted similar calculation method to the one reported by Son *et al*.[^2^](#_ENREF_2) to evaluate the strain introduced by the van der Waals interaction between the stuck portion of nanotube and the trench bottom surface.

The model is based on a molecular wire bound to a substrate with a contact angle $\alpha$. With the configuration illustrated in Fig. S3a, the strain that is generated to the SWNT is given by:

$\varepsilon\text{=}\frac{T}{AY}=\frac{E_{B}}{AY}\frac{1}{1-\cos\alpha}$ (S1)

where $T$ is the tension induced to the SWNT, $A$ is the cross section area of the nanotube, $Y$ is Young’s modulus of SWNT, which lies close to 1 TPa[^3^](#_ENREF_3), and $E_{B}$ is binding energy per unit length and is around 0.8 eV/Å[^4^](#_ENREF_4). The contact angle $\alpha$ can be measured from SEM images of SWNTs crossing trenched structures. An exemplary observation is displayed in Fig. S3b, where an individual SWNT is lying across a 20 µm wide and 2 µm deep trench. It can be seen that approximate 6 µm long of the SWNT portion is in contact with the trench bottom, thus the contact angle, as pointed out in Fig. S3a, is estimated to be 16°.

We measured SWNTs spanning different trenched structures, and obtained the contact angle in the range of 14° to 22°. By using equation S1 and taking the SWNT diameter as 1.9 nm, we calculated the strain induced by the “tube-lower substrate adhesion effect” ranging from 0.4% to 1.2%.

**Electrical measurements on as-prepared S-S IMJ.**

The electrical properties of the as-fabricated SWNT IMJ were probed by assembling the nanostructure into a field-effect transistor (FET) configuration, as depicted in the inset of Fig. S4a. For comparison, another FET was fabricated based on the adjacent SOS segment of the same individual SWNT, which exhibits the intrinsic electrical properties of the semiconducting nanotube. Adjacent SOS segment and SUS segment (including the trench edges) from the same individual SWNT were covered with arrays of Ti/Au (10/60 nm in thickness) electrodes (serving as source and drain electrodes) using photolithography technique and electron beam deposition. The n-doped silicon wafer was used as the gate electrode, while the SiO_2_ layer acted as the gate dielectric. The length of SWNT segment in between any electrode pair was kept constant. The electrical measurements were carried out in air at room temperature by an Agilent 4156B semiconductor parameter analyzer.

Typical $I_{\text{ds}}-V_{\text{ds}}$ (drain-source current versus drain-source bias) characteristics of the SOS and SUS segments are compared in Fig. S4a (obtained at -16 V gate voltage, $V_{\text{g}}$ ). A symmetric $I_{\text{ds}}-V_{\text{ds}}$ curve is observed from the SOS segment, while the SUS segment shows an asymmetric nonlinear feature, similar to that of a rectifying diode. The conductance of both the SOS and SUS segments could be drastically varied with the change of the applied gate voltage values, as shown in Fig. S4b and S4c. The transfer curves ($I_{\text{ds}}-V_{\text{g}}$) of the two segments were measured at a constant drain-source bias of 2 V and displayed in Fig. S4d. The on-off current ratio is increased by nearly one order of magnitude from the parent SWNT to the S-S junction. We may thus infer that the band structure of the nanotube was tuned at the interface of the two segments, forming a barrier across the IMJ, thereby resulting in the rectifying behavior[^5^](#_ENREF_5). The unique properties of the IMJs enable the fabrication of diodes in a controlled and large-scale fashion. Furthermore, the suspended SWNT devices permit the coupling of electron tunneling with mechanical motion, holding great promises in applications of nanoelectromechanical systems (NEMS) .

**Study of $\omega_{G^{+}}$ shift and $I_{D}/I_{G}$ ratio variation in the case of other trenched structures**

By taking the striking downshift of $\omega_{G^{+}}$ and drastic variation of $I_{D}/I_{G}$ ratio along the tube axis as evidence of formation of IMJs, we studied the rest of the specific nanotube across other trenches and also different ultralong SWNTs across trenches. In all cases, we chose wide and shallow trenched structures (20 µm wide and 1~3 µm deep) to ensure the SWNTs are partially suspended across the trenches with middle portion adhered to trench bottom. With this effort, we investigated the formation of IMJs in the trans regions, due to the mechanical deformations. Four typical results are exemplified in Fig. S5.

**References**

1 Zhang, Y., Son, H., Zhang, J., Kong, J. & Liu, Z. Laser-heating effect on Raman spectra of individual suspended single-walled carbon nanotubes. *J. Phys. Chem. C* **111**, 1988-1992 (2007).

2 Son, H. *et al.* Strain and friction induced by van der Waals interaction in individual single walled carbon nanotubes. *Appl. Phys. Lett.* **90**, 253113 (2007).

3 Lu, J. P. Elastic properties of carbon nanotubes and nanoropes. *Phys. Rev. Lett.* **79**, 1297-1300 (1997).

4 Hertel, T., Walkup, R. E. & Avouris, P. Deformation of carbon nanotubes by surface van der Waals forces. *Phys. Rev. B* **58**, 13870-13873 (1998).

5 Yao, Y. *et al.* Temperature-mediated growth of single-walled carbon-nanotube intramolecular junctions. *Nat. Mater.* **6**, 283-286 (2007).

6 Häkkinen, P., Isacsson, A., Savin, A., Sulkko, J. & Hakonen, P. Charge sensitivity enhancement via mechanical oscillation in suspended carbon nanotube devices. *Nano Lett.* **15**, 1667-1672 (2015).

7 Bushmaker, A. W. *et al.* Single-ion adsorption and switching in carbon nanotubes. *Nat. Commun.* **7**, 10475 (2016).
